# Epidemiology of Penetrating Eye Injury in Ibadan: A 10-Year Hospital-Based Review

**DOI:** 10.4103/0974-9233.80706

**Published:** 2011

**Authors:** Fasina Oluyemi

**Affiliations:** Department of Ophthalmology, University College Hospital, Ibadan, Oyo State, Nigeria

**Keywords:** Eye Injury, Ocular Injury, Ocular Trauma, Penetrating Ocular Injury

## Abstract

**Purpose:**

To assess risk factors associated with the occurrence of penetrating ocular injuries among patients presenting to an eye hospital at Ibadan, Nigeria.

**Materials and Methods:**

All cases of penetrating ocular injury presenting over a 10 - year period, were identified by retrospective chart review. All current cases of penetrating ocular injury identified were included. All information was obtained retrospectively from the medical records.

**Results:**

The cohort consisted of 135 cases. The follow-up was for an average period of 24.6 weeks (range, 12-312 weeks). Injuries were most likely to occur at home, in a domestic setting (58%). The most common mechanism of injury was projectile missiles hitting the eye. The age range for injuries was 9 months to 70 years. Penetrating ocular injury was most frequent in the 20-29 years group (31.9%) followed by the 0–9 years age group (31.1%). Males were more frequently involved than females (ratio 4:1). The final acuity was better than 6/18 in 14.8% and less than 3/60 in 59.3% of cases.

**Conclusions:**

Penetrating ocular injury occurs, most frequently, in a domestic setting and mostly as a result of working with sharp objects. Preventive measures are recommended to reduce visual disabilities due to ocular injuries.

## INTRODUCTION

Ocular trauma is an important cause of preventable morbidity worldwide, and is a major cause of unilateral visual loss in developing countries.^1^,^2^ The epidemiology has been studied in developed countries^3^–^5^ and some developing countries.^6^–^8^ Data on ocular injuries in Nigeria is available from previous studies.^9^–^13^ However, the pattern of ocular injury in the country can be influenced by changes in the environmental and socio-economic lifestyle and government policies.For example, a shift in occupation from predominantly agricultural to urban, the emerging ethno-religious strife and recently, the enforcement of seat belts usecan affect the pattern of penetrating eye injuries in the country.

Risk factors associated with ocular trauma include gender, age, occupation, and lower socio-economic status.^2^,^14^ The socio-economic impact of penetrating eye injuries is tremendous in a developing country, like Nigeria, given the fact that the patient invariably requires hospital admission and surgical intervention.^15^ This epidemiologic review studies the current pattern of penetrating ocular injuries in patients who presented to a tertiary eye care in Nigeria, and focuses on identifying etiology, risk factors, and possible preventive measures.

## MATERIALS AND METHODS

A chart review was performed of patients with penetrating eye injuries between January 1998 and December 2008, based eye clinic emergency register, ward admissions register and theater operations register of the Department of Ophthalmology, University College Hospital, in Ibadan, Nigeria Penetrating eye injury in this study was defined as an open globe injury caused by a sharp object, with or without a retained intraocular foreign body.^16^ Ibadan is the capital city of Oyo State located in the South Western region of Nigeria. The ophthalmology department ofthe hospital, a tertiary health facility, serves as a major referral center for specialized eye care in Ibadan and the surrounding towns and villages. Agriculture is the predominant occupation of the people living in the surrounding towns and villages. Hence, patients from all socioeconomic strata access the eye care services of the hospital. This is indicative of most tertiary health care facilities in Nigeria, which are located in large cities and treat patients referred from surrounding semi-urban and rural towns and villages. Patients are treated in the eye unit through referral, although, emergency cases do not warrant referral.

Information collected for this study included age, sex, occupation, cause of injury, duration of injury before presentation, activity leading to injury, type of injury, unilateral or bilateral injury, vision at presentation, treatment given, complications of the injury, and best corrected vision at the last follow-up visit. Complete data were extracted from all the three registers. All cases of penetrating eye injury required admission and/or surgery, resulting in well-documented charts without significant data omission. This study was approved by the Ethical Review Board of the hospital and follows the tenets of Declaration of Helsinki.

Statistical analyses were performed using the statistical packages for the social sciences version 15 (SPSS, Inc., Chicago, IL, USA). Frequency and percentages were used to report categorical variables while mean, median, and standard deviations were used in reporting quantitative variables. A *P* value less than 0.05 was considered statistically significant.

## RESULTS

A total of 146 patients with penetrating eye injuries were identified based on chart review. One hundred and thirty-five patients had had complete data in their case records, and were analyzed. There were 108 (80%) males with a male to female ratio of 4:1 (*P* = 0.697). The patients ranged in age from 9 months to 70 years with a median of 18.0 years. Fifty-six patients (41.5%) were aged less than 15 years old [Table 1]. The mean follow-up was 24.6 weeks (range, 12 - 312 weeks).

**Table 1:** Age group of the cases with penetrating eye injury

Age group (years)	Frequency	Percentage	(95% CI)
0-9	42	31.1	(23.2-39.0)
10-19	28	20.7	(13.8-27.7)
20-29	43	31.9	(23.8-39.8)
30-39	14	10.4	(5.2-15.6)
40+	8	5.9	(1.9-9.9)
Total	135	100	

The right eye was affected in 45.9% of patients. There were no bilateral cases. Wounds were predominantly corneal (43.7%) or corneo-scleral (41.5%). Uveal prolapse occurred in 68.1% of the patients, 47.4% of the patients presented with hyphema, and 28.1% presented with cataract [Table 2]. Only 2 patients (1.5%) had no associated injury with their corneal laceration.

**Table 2:** Other ocular manifestations in eyes with penetrating injuries

Injury*	Frequency	Percentage
Uvea prolapsed	92	68.1
Hyphema	64	47.4
Cataract	38	28.1
Lid laceration	19	14.1
Endophthalmitis	4	3.0
Intra ocular foreign body	6	4.4
Others	11	8.1
Nil	2	1.5

* More than one option possible

**Figure 1:**
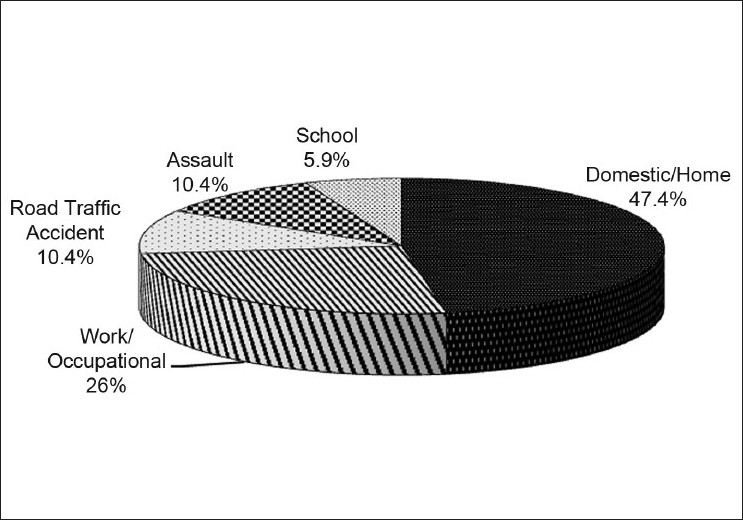
Type/setting of penetrating ocular injury

Ocular trauma occurred in a domestic setting in 64 (47.4%) cases. Injuries at school were infrequent (5.9%) [Figure 1].

The most common mechanism of injury was from a chip of metal or vegetative matter impacting the eye. This occurred accidentally in a domestic setting when individuals were handling sharp objects and carrying out minor repair-work [Table 3]. Injuries in children (less than 15 years), were mostly due to playing with sharp objects (pencil tip, metal, and wooden pieces)

**Table 3:** Causes of penetrating ocular injuries in Ibadan

Cause(s)	Frequency	Percentage	(95% CI)
Shattered glass/bottle	29	21.5	(14.5-28.5)
Whiplash/stick/vegetative matter	20	14.8	(8.7-20.9)
Missile (chip of nail/instruments)	19	14.1	(8.1-20.0)
Sharp tip of metallic objects	16	11.9	(6.3-17.4)
Sharp edge of blade/knife/cutlass	12	8.9	(4.0-13.8)
Gunshot	9	6.7	(2.4-10.9)
Missile (stone)	8	5.9	(1.9-9.9)
Fall	8	5.9	(1.9-9.9)
Sharp tip of pencil/ball pen/needle	6	4.4	(0.9-7.9)
Others	8	5.9	(1.9-9.9)
Total	135	100.0	

Seventy-two (53.3%) patients were seen within 24 h of the injury, whereas 39 (28.9%) patients presented after 72 h. Presenting visual acuity was better than 6/18 in only 4 (3.0%) patients, whereas 85 (63.0%) patients had acuity less than 3/60 at presentation. Twenty-two (16.3%) patients had no light perception at presentation. At last follow-up, the overall best corrected visual acuity (BCVA) was: 20 (14.8%) patients with 6/18 or better BCVA; 80 (59.3%) patients with 3/60 or less BCVA; and, 34 (25.2%) patients had no light perception [Table 4]. The various causes of poor vision (<3/60) are presented in Table 5. All patients had corneal opacities, and fifty-six (70%) patients had a phthisical eye.

**Table 4:** Visual acuity at presentation and last follow up of penetrating ocular injury cases

VA	At presentation		At last follow up		*P* value
	Frequency	Percentage (95% CI)	Frequency	Percentage (95% CI)	
>6/18	4	3.0 (0.01-5.9)	20	14.8 (8.7-20.9)	
6/18-3/60	16	11.8 (6.3-17.4)	26	19.2 (12.5-25.9)	
<3/60	85	63.0 (54.7-71.2)	80	59.3 (50.8-67.6)	0.000
Not possible	30	22.2 (15.1-29.3)	9	6.7 (2.4-10.9)	
Total	135	100.0	135	100.0	

VA: Visual acuity

**Table 5:** Causes of poor visual outcome (<3/60) following management of penetrating ocular injuries

Cause*	Frequency	Percentage
Corneal opacity	80	100
Phthysis bulbi	56	70
Cataract	28	35
Retinal detachment	4	5
Anterior staphyloma	1	1.3
Ciliary staphyloma	1	1.3
Vitreous hemorrhage	1	1.3

* More than 1 cause possible

## DISCUSSION

Ocular injury is a well-established cause of preventable visual loss in youngindividuals.^17^–^19^ The epidemiology of ocular injuries varies from community to community, region to region and with time.^19^–^21^

In this study, there was a higher, yet statistically insignificant, incidence of penetrating ocular injuries among males, though compared to females (*P* = 0.697). The male to female ratio was 4:1, which is consistent with findings from the majority of similar studies.^1^,^6^,^7^,^9^,^22^–^24^ However, this ratio was slightly higher than previous studies^12^,^22^,^25^ in the Ibadan area. The discrepancy is due to the greater variety of ocular trauma evaluated in previous studies.^12^,^22^,^25^ The fact that more males are involved in high-risk behavior and vocation, and are adventurous and aggressive makes them more prone to ocular injuries.^22^ Male children also exhibit greater mobility and a higher incidence of violent outdoor activities.^26^ Similar to other studies,^11^,^12^,^22^,^27^ the majority (83.7%) of individuals affected by penetrating eye injuries in our study are children and adults during an active age (<30 years).

The majority of the injuries in the current study occurred as household accidents, followed by work-related injuries. Similar findings have been reported in previous studies.^9^,^18^,^28^,^29^ Our observations, however, differ from those of Okoye^22^ and Adeoye,^30^ who reported assault/combat was the major cause of ocular injuries. The difference in observation between the current study and previous studies^22^,^30^ indicates a variation in the etiology of eye injuries at different times and localities. The low prevalence of injuries following road traffic accidents in our series (10.4%) can be attributed in part to enforcement seat belt use as previously reported.^31^,^32^ A large proportion of these injuries occurred under domestic setting and perhaps preventive measures in houses could reduce such ocular injuries in the future. Similar to the findings of Luff *et al*.,^33^ a few cases (5.9%) of ocular trauma occurred in school in our series. Injuries occurring in the school are more likely to be non-perforating,^33^ and thus, were also excluded from our study.

The cornea, and corneo-scleral regions were the most common sites of injury due to the greater exposure of these structures to impact.^18^ More than half (53.3%) of the injuries resulted from projectile objects (shattered glass, metallic and non-metallic missiles, gunshot, and so on), and the remaining 46.7% resulted from sharp-tipped objects penetrating the eye (sharp-tipped metallic and non-metallic objects, sharp-edged instruments, falls, and so on). Uveal prolapse was common in the majority of cases of ocular trauma.

Only half of the cohort presented at eye clinic within 24 h of injury. Late presentation of patients with ocular trauma has been well reported in previous studies.^9^,^11^,^19^,^29^,^34^ Bekibele *et al*.,^27^ in their study, however, reported that 75% of their patients reported within 24 h of injury. This might be explained by the importance placed on injuries caused purely by high-velocity missiles. Delayed presentation leads to prolonged inflammation and a greater risk of infection with tissue disorganization in these patients.^10^,^34^ A concerted effort to educate, the public, primary health care providers, and other health care personnel on the importance of timely referral and timely treatment of ocular injury is warranted.

The majority of patients (63%) in the current study had presenting visual acuity less than 3/60. This is not surprising, as penetrating injuries have worse prognosis compared with concussion injuries.^12^,^22^ At last follow-up, overall visual acuity of better than 6/18 was achieved in 14.8% of our patients. However, about 60% still had acuity less than 3/60 and a quarter had no light perception. Our outcomes are similar to previous studies of penetrating/perforating eye injuries,^22^,^34^,^35^ and demonstrate the severity and the resulting visual morbidity due to penetrating ocular trauma.

There are some strengths and limitations of this study. By retrieving data from three sources- eye clinic emergency register, ward admissions register, and theater operations register, an almost complete list of all cases of penetrating ocular injuries was collected and analyzed. However, due to the retrospective nature of the study, some patients who did not consent to admission and surgical repair have been excluded. Eleven case records with incomplete data had to be excluded from analysis. However, we believe this is a representative sample of ocular trauma in the Ibadan region.

Since prevention is the goal in the management of penetrating eye injuries, greater attention should be directed to potential causes of injury at home and the workplace. Majority of the injuries, from our study, occurred in a domestic setting. Hence, we recommend wearing protective eye gear, while engaged in potentially dangerous tasks, not only at work but also at home. The public should be encouraged to wear adequate protection regardless of where the potentially dangerous activity is being performed. Adequate supervision of children must be emphasized, and using or playing with sharp tools and toys should be discouraged. The enforcement of seat belt use should also be a sustained effort.

## References

[1] Thylefors B (1992). Epidemiological patterns of ocular trauma. Aust N Z J Ophthalmol.

[2] Negrel AD, Thylefors B (1998). The global impact of eye injuries. Ophthalmic Epidemiol.

[3] Fong LP (1995). Eye injuries in Victoria, Australia. Med J Aust.

[4] Tielsch JM, Parver LM, Shankar B (1989). Time trends in the incidence of hospitalised ocular trauma. Arch Ophthalmol.

[5] Blomdahl S, Norell S (1984). Perforating eye injury in the Stockholm population. Acta Ophthalmol.

[6] Gothwal VK, Adolph S, Jalali S, Naduvilath TJ (1999). Demography and prognostic factors of ocular injuries in South India. Aust N Z J Ophthalmol.

[7] Ilsar M, Chirambo M, Belkin M (1982). Ocular injuries in Malawi. Br J Ophthalmol.

[8] Khan MD, Kundi N, Mohammad Z, Nazeer AF (1988). Eye injuries in the North West Frontier Province of Pakistan. Pak J Ophthalmol.

[9] Olurin O (1971). Eye injuries in Nigeria. Am J Ophthalmol.

[10] Ajayi BG, Osuntokun O (1986). Perforating eye injuries in Ibadan. West Afr J Med.

[11] Onabolu OO (1994). Visual loss in ocular trauma. Niger J Ophthalmol.

[12] Ajaiyeoba AI (1995). Ocular injuries in Ibadan. Niger J Ophthalmol.

[13] Umeh RE, Umeh OC (1997). Causes and visual outcome of childhood eye injuries in Nigeria. Eye.

[14] Dandona R, Dandona L (2003). Corneal blindness in a southern Indian population: Need for health promotion strategies. Br J Ophthalmol.

[15] Landen D, Baker D, LaPorte R, Thoft R (1990). Perforating eye injury in Allegheny county, Pennsylvania. Am J Public Health.

[16] Pieramici DJ, Sternberg P, Aaberg TM, Bridges WZ, Capone A, Cardillo JA (1997). A system for classifying mechanical injuries of the eye (globe).The Ocular Trauma Classification Group. Am J Ophthalmol.

[17] Negrel AD (1997). Magnitude of eye injuries worldwide. Community Eye Health.

[18] Thompson CG, Griffits RK, Nardi W, Tester MP, Noble MJ, Cottee L (1997). Penetrating eyeinjuries in rural New South Wales. Aust N Z J Ophthalmol.

[19] Shah M, Shah S, Khandekar R (2008). Ocular injuries and visual status before and after their management in the tribal areas of Western India-A historical cohort study. Graefes Arch Clin Exp Ophthalmol.

[20] Canavan YM, O’Flaherty MJ, Archer DB, Elwood JH (1980). A 10-year survey of eye injuries in Northern Ireland, 1967-76. Br J Ophthalmol.

[21] Krishnaiah S, Nirmalan PK, Shamanna BR, Srinivas M, Rao GN, Thomas R (2006). Ocular trauma in a rural population of southern India: The Andhra Pradesh Eye Disease Study. Ophthalmology.

[22] Okoye OI (2006). Eye injury requiring hospitalisation in Enugu Nigeria: A one-year survey. Niger J Surg Res.

[23] Cillino S, Casuccio A, Di Pace F, Pillitteri F, Cillino G (2008). A five-year retrospective study of the epidemiological characteristics and visual outcomes of patients hospitalized for ocular trauma in a Mediterranean area. BMC Ophthalmol.

[24] Soliman MM, Macky TA (2008). Pattern of ocular trauma in Egypt. Graefes Arch Clin Exp Ophthalmol.

[25] Adefule-Ositelu AO, Soetan II, Akinsola FB (1996). Ocular trauma in Lagos. West Afr J Med.

[26] Jaison SG, Silas SE, Daniel R, Chopra SK (1994). A review of childhood admission with perforating ocular injuries in a hospital in north-west India. Indian J Ophthalmol.

[27] Bekibele CO, Ajayi BG, Baiyeroju AM, Ayeni EA (2003). Visual outcome of pressurised bottled drinks related eye injuries in Ibadan. Afr J Med Med Sci.

[28] Oshoba FO (1994). Ocular trauma in Lagos. Niger J Ophthalmol.

[29] Abiose A (1975). Eye injuries in Lagos. Niger Med J.

[30] Adeoye AO (1996). Eye injuries caused by locally manufactured dane guns. Niger J Ophthalmol.

[31] Elder M (1993). Penetrating eye injuries in children of the West Bank and Gaza strip. Eye (Lond).

[32] Thompson CG, Kumar N, Billson FA, Martin F (2002). The aetiology of perforating ocular injuries in children. Br J Ophthalmol.

[33] Luff AJ, Hodgkins PR, Baxter RJ, Morrel AJ, Calder I (1993). Aetiology of perforating eye injury. Arch Dis Child.

[34] Baiyeroju-Agbeja AM, Olurin-Aina OI (1998). Penetrating eye injuries in children in Ibadan. Afr J Med Med Sci.

[35] Niiranen M, Raivio I (1981). Eye injuries in children. Br J Ophthalmol.

